# Effects of 3D Printing-Line Directions for Stretchable Sensor Performances

**DOI:** 10.3390/ma14071791

**Published:** 2021-04-05

**Authors:** Chi Cuong Vu, Thanh Tai Nguyen, Sangun Kim, Jooyong Kim

**Affiliations:** Department of Organic Materials and Fibers Engineering, Soongsil University, Seoul 156-743, Korea; cuongvc287@gmail.com (C.C.V.); ntt16895@gmail.com (T.T.N.); tkddnsl0723@naver.com (S.K.)

**Keywords:** 3D printing, stretchable sensors, printing-line direction, thermoplastic polyurethane

## Abstract

Health monitoring sensors that are attached to clothing are a new trend of the times, especially stretchable sensors for human motion measurements or biological markers. However, price, durability, and performance always are major problems to be addressed and three-dimensional (3D) printing combined with conductive flexible materials (thermoplastic polyurethane) can be an optimal solution. Herein, we evaluate the effects of 3D printing-line directions (45°, 90°, 180°) on the sensor performances. Using fused filament fabrication (FDM) technology, the sensors are created with different print styles for specific purposes. We also discuss some main issues of the stretch sensors from Carbon Nanotube/Thermoplastic Polyurethane (CNT/TPU) and FDM. Our sensor achieves outstanding stability (10,000 cycles) and reliability, which are verified through repeated measurements. Its capability is demonstrated in a real application when detecting finger motion by a sensor-integrated into gloves. This paper is expected to bring contribution to the development of flexible conductive materials—based on 3D printing.

## 1. Introduction

Three-dimensional (3D) printing enables us to create complex shapes using fewer materials than traditional manufacturing methods [1,2,3]. In which, fused deposition modeling (FDM) is a 3D printing process that uses a continuous filament from thermoplastic materials [4,5,6]. The filament is heated and extruded by a nozzle. Each printed layer is laid down one at a time until the object is complete. This method has become popular, because it provides a cleaning process, easy-to-use, and cost-effective. Nowadays, stretchable sensors, which are fabricated by 3D printing, have demonstrated important roles during the evolution of electronic skin [7,8], wearable electronics [9,10,11], and biomedicine [12,13].

When considering flexible parts created by 3D printing, thermoplastic polyurethane (TPU) is well known. TPU possesses high elasticity (as rubber), tear or abrasion resistance, high elongation, as well as thermal stability [14,15,16]. When containing conductive components (carbon nanotube, CNT), CNT/TPU is definitely a highlight material to fabricate the stretch sensors [17,18,19,20]. Following this approach, there are many great studies in the stretch sensing field, which are enhanced with advanced materials based on FDM printing [21,22,23,24,25]. For example, Josef et al. [26] developed 3D printed highly elastic strain sensors of multiwalled carbon nanotube/thermoplastic polyurethane (MWCNT/TPU) nanocomposites. Gauge factors achieve ~ 176. Dong et al. [10] presented CNT/TPU nanocomposites via non-covalent interactions to enhance the performance of the stretch sensors. These 3D sensors demonstrated excellent properties with a high gauge factor and large detectable strain (250%). In addition, Saeb et al. [27] developed high anisotropic, flexible, constriction-resistive sensors. The sensing elements and conductive interconnects are 3D-printed from a carbon-nanotube-reinforced polylactic acid (PLA-CNT). The sensor parameters can be adjusted by controlling the air gap between printed adjacent tracks, infill density, and build orientation. However, the characteristics of the printing-line directions have not been discussed in depth.

Here, we focus on the one-step processing of stretchable sensors using the TPU material and FDM method. Especially, the effects of printing-line directions (45°, 90°, 180°) on sensor performances are evaluated in detail. The sensor types are printed by a rebuilt RepRap Prusa I3 3D printer, which is an ideal option for a low-cost FDM 3D printer. The printing parameters are set up and the thickness of the sensors is about 0.4 mm. We observe that the printing direction at 45° is the best solution to enhance the characteristics of the sensor. Besides, the sensor is integrated into gloves to detect finger motions. This prototype application explains the potential of our sensor in smart garments.

Some previous studies on the effects of geometry (shapes and structures) on the strain sensors [28] and 3D printing samples [29,30,31] are the motivation for the paper. We realize that the geometric approach is easy, fast, and low-cost to improve sensor performance in wearable systems. Accordingly, this research is expected to be a standardization for 3D-printing strain sensors.

## 2. Materials and Methods

### 2.1. Materials

Conductive flexible filament (TPU/CNT) was acquired from Black Magic 3D. The diameter of CNTs (PI-ETPU, Palmiga Innovation, Jonstorp, Sweden) is about 9.5 nm and the length of CNTs in the TPU filaments is about 3 µm, according to information from the product supplier. The characteristics of TPU can ensure flexibility and good compatibility with 3D printing. The conductivity of the filament is under 1.25 Ω-cm. The diameter is 1.75 mm and tolerance is +/− 0.03 mm. 3D FDM printer (RepRap Prusa I3) parts were prepared from Naserpop Com., Incheon, South Korea.

### 2.2. Printing Method

Three types of samples are designed in a rectangle shape on Solidworks software (version 2019, Solidworks Corp., Waltham, MA, USA) and then processed via Repetier-Host software (version 2.1.6, Hot-World GmbH & Co. KG, Willich, Germany) with a dimension of 4 mm × 60 mm, and a thickness of 0.4 mm. Before printing, the conductive flexible TPU filament is dried at 70 °C for four hours in order to remove moisture. This procedure helps the printed samples have better resistance. These sensors are constructed in three different types at the printing-line directions (45°, 90°, 180°) with a nozzle size of 0.2 mm. Each sample will be printed in two layers. The thickness of one layer is 0.2 mm. This makes the printing is easy; the sensors are thin and stretchable.

**Figure 1:**
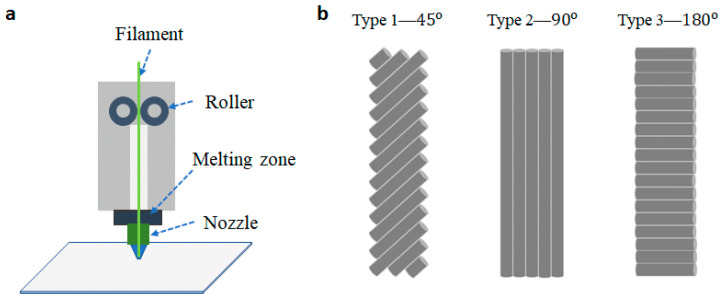
(**a**) Fused deposition modeling (FDM), (**b**) three structures (45°, 90°, 180°) of three-dimensional (3D) printing-line directions.

TPU is flexible. Hence, the filament is easy to get stuck or flat between the rollers (as shown in Figure 1a), owing to heat conduction [30,31,32]. Herein, the heat transfer from one end to another of the distance (L) of TPU filament can be considered as heat conduction. Equation (1) describes this process.

(1) $$\frac{Q}{t}=\frac{k\;*\;A\cdot \left(T_{\mathrm{hot}}-T_{\mathrm{cold}}\right)}{L}$$

where Q is the heat transfer in time, k is the thermal conductivity of TPU, A is the area of cross-section filament, T is the temperature, and L is the distance from the hot end to the cold end of the filament.

Some of the solutions can be used to avoid the filament sticking issue, for example, increasing the area of the cross-section of extending the distance (L) by high flow rate (the rate of plastic exiting the extruder’s nozzle) or decreasing the printing temperature. Herein, we set up the temperature at 230 °C in order to minimize the possibility of blockages and the appearance of air bubbles in the samples. In addition, the speed of the extruder head is another important parameter to stop or start deposition. This value (at the speed of 20 mm/s) will create stable printing lines without interruption.

The FDM method presents a disadvantage when using available conductive filaments (CNT/TPU). Heat treatment/print will generate many spaces between the conductive particles, and the concentration of the CNT per sample reduces. This leads to a significant increase in the resistance of the sensors. These issues can be resolved by decreasing the temperature or changing the material (increasing the rate of CNTs). However, this method also increases the manufacturing-cost and it needs more experimentations.

## 3. Results and Discussion

### 3.1. Structure of the Sensors

Figure 1a shows the process of the fused deposition modeling (FDM) in 3D printing technology. Conductive filament (CNT/TPU) is fed from a large spool through a moving, heated printer extruder head, and it is deposited on the growing work. The position and speed of the extruder head will move under the control of a computer to define the sensor shapes. Melting temperature and extrusion speed are two main parameters, which define the quality of the printing surface. There are three types due to the direction of the printing-lines at 45°, 90°, and 180°, as described in Figure 1b.

**Figure 2:**
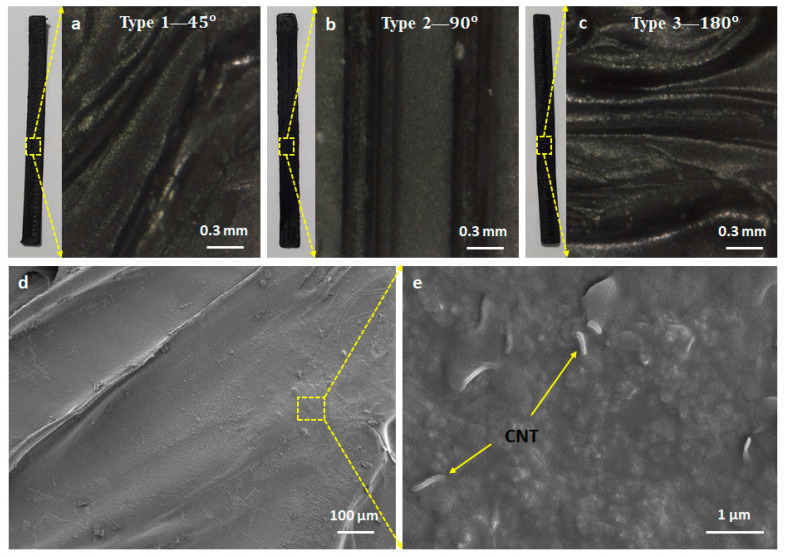
Scanning electron microscope (SEM) images of the sensors, consisting of (**a**) Type 1—45° at 0.3 mm, (**b**) Type 2—90° at 0.3 mm, (**c**) Type 3—180° at 0.3 mm, (**d**) SEM of the printing line at 100 µm, and (**e**) SEM of the printing line at 1 µm.

Using scanning electron microscopy (Gemini SEM 300, ZEISS Corp., Jena, Germany), the magnified images are shown in Figure 2, at 0.3 mm, 100 µm, and 1 µm, respectively. We observe that CNTs are about 9.5 nm in diameter and homogeneously dispersed in TPU filament. The majority of these carbon nanotubes are fractured under strain/release cycles, inducing the resistance change of the sensors.

### 3.2. Characteristics of the Sensors

**Figure 3:**
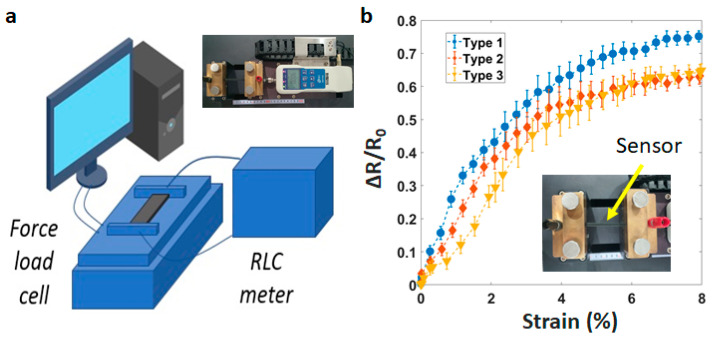
(**a**) Universal testing machine (UTM), (**b**) Sensitivity of the sensors with type 1—45°, type 2—90°, type 3—180°.

In order to analyze the fabricated sensor’s electrical characteristics, we develop an experiment with a customized universal testing machine. As shown in Figure 3a, this system includes a force load cell from Dacell Co. Ltd., Seoul, Korea, a source meter (Keysight B2902A), and a computer. The sensitivity of sensors is demonstrated through the gauge factor (Equation (2)).

(2) $$\mathrm{GF}\;=\;\frac{∆R/R}{\varepsilon }=\frac{∆R/R}{∆L/L}$$

where GF is the gauge factor, R is the initial resistance, ∆R is the absolute change in resistance, L is the initial length, and ∆L is the absolute change in length.

Five samples of each type are tested and averaged at the strain speed of 1 mm/s. The largest sensitivity is the printing line at 45° (Figure 3b). GF is about 21.5 when stretching from 0–2%, and 16.2 when stretching from 2–4%. However, it drops to 10 when stretching from 4–8%. For other printing lines (90° and 180°), GF is about 12.5 when stretching from 0–4%, and 5.5 when stretching from 4–8%. The acceptable performance of a strain sensor is determined by the ability to detect changes in structure under minimum deformation. This parameter is shown significantly in the sensitivity (GF). Actually, all three types of our sensors (45°, 90°, 180°) demonstrate good performances when compared to the samples of Christ et al. with GF ~ 3.0 [33], Rahman et al. with GF ~ 3.15 [34], or Yao et al. with GF ~ 6.6 [35].

**Figure 4:**
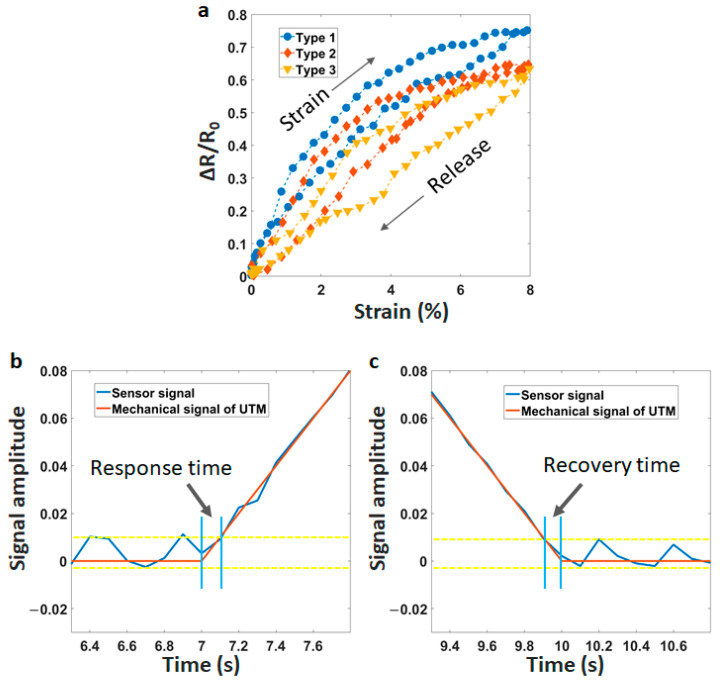
(**a**) Hysteresis of the sensors with type 1—45°, type 2—90°, type 3—180°, (**b**) Response time, and (**c**) Recovery time.

Hysteresis represents the historical dependence of a system. It is often evaluated by the difference in the sensor signals at two phases (strain and release) of a cycle. We investigate the hysteresis of sensors in Figure 4a. There is a significant difference between the printing-line direction at 45° and the two others. The maximum hysteresis is under 10% for line at 45°. Meanwhile, the value is over 30% for lines at 90° and 180°.

The response time refers to the time span between the sensor signal and the mechanical stimulation of the universal testing machine (UTM). The response time can be estimated as 120 ms in the increasing step of the tension, as described in Figure 4b. Besides, the recovery time is defined as the time for the sensor to return to the baseline value after removing tension. Figure 4c shows a short recovery time of less than 100 ms. The delay time is caused by the viscoelastic nature of TPU and the re-connectivity between CNT-particles under strain force. A fast response/recovery time will ensure the rapid electrical property in a practical application. Based on those results, the printing-line direction at 45° shows a good solution in real applications when creating the stretch sensors from FDM technology.

**Figure 5:**
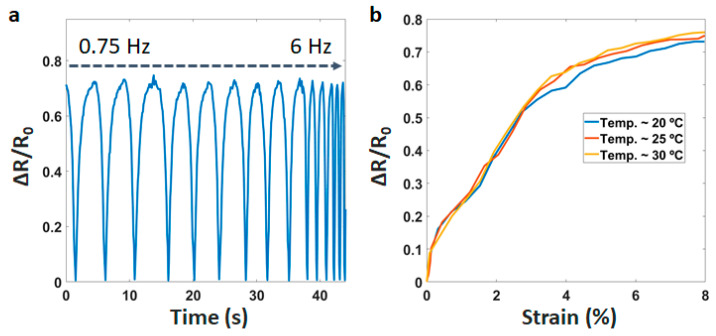
(**a**) Sensitivity of the sensor at different frequencies, (**b**) Resistance changes at different temperatures.

We also investigate the dynamic performance of the sensor (Figure 5a). It is clear that the sensor has a stable response under a wide mechanical frequency range from 0.75 Hz to 6 Hz. Most of the wearable devices work around room temperature (~25 °C). Therefore, the operation of the sensor is tested in the temperature range of 20 to 30 °C. This will ensure that the sensor potential is in a realistic application. After testing, Figure 5b describes the effect of temperature on the performance. We observed that the resistance has a little change (<7%) in the temperature range of 20 to 30 °C. This result is achieved due to the filament being processed through the melting step. Accordingly, the sensor is less affected by the temperature.

**Figure 6:**
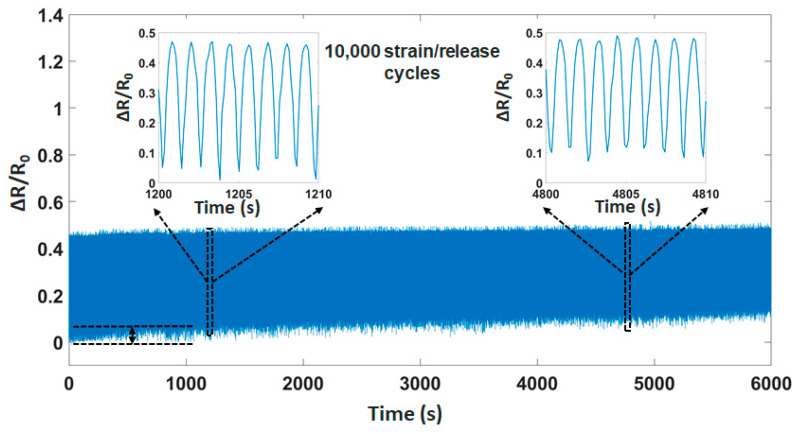
Durability of the sensor in 10,000 strain/release cycles.

Figure 6 describes the stable electrical functionality and mechanical integrity during multiple strain/release cycles (based on the strain force of UTM machine). We can observe that the durability of the sensor is over 10,000 cycles at the frequency of 2 Hz. There is a small difference at the limit of sensing ~8%. The reason for this behavior is the deformation (permanent) in the structure under pressure, leading to a small change in the sensing limitation.

### 3.3. Applications

**Figure 7:**
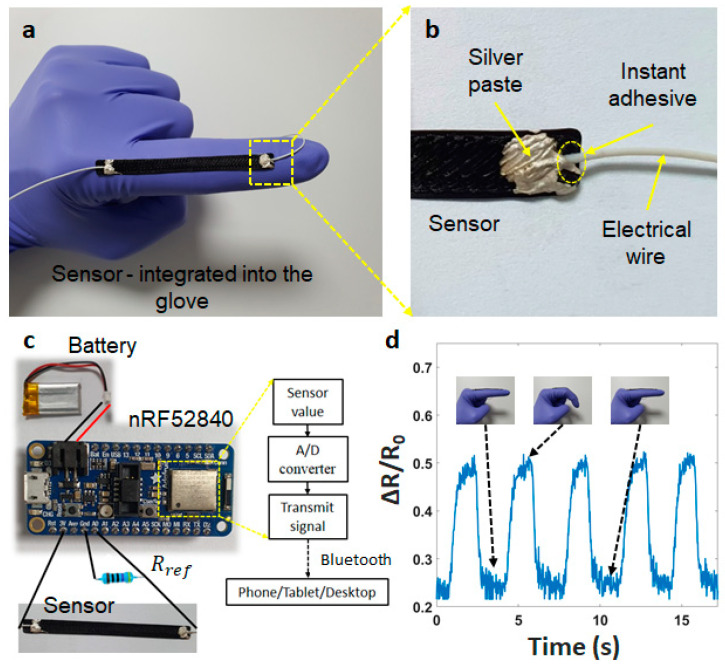
(**a**) Structure of the sensor integrated into the glove, (**b**) Structure of the connection, (**c**) Integrated device, and (**d**) Resistance changes of the finger when bending at 45° and recovering.

The sensor is attached to a glove for tracking finger motions to demonstrate the potential applications (Figure 7a). The electrical wires (AWG 32) are fixed on both edges of the sensor using instant adhesives and stretchable silver pastes, as shown in Figure 7b (from Dycotec Materials Ltd., Calne, UK). The signal processing system is an integrated circuit (Figure 7c), consisting of an nRF52 module (analog/digital converter—ADC, microcontroller unit—MCU, Bluetooth) and a lipo-battery (3.7 V). Using a voltage divider circuit, the analog signals are sampled/digitized and converted into digital signals. The resolution is set up between 0 to 3 V into digital values from 0 to 1023 (3/1023 ~ 0.003 V or 3 mV per unit). The input signal is read every 10 ms.

The finger movement is tested when increasing the bending angle from 0 to 45° (Figure 7d). The sensor signal immediately increases following the bending angle, but makes a slight noise at the start and the end phases. This can be explained by the elastic features of the glove material, which makes it difficult to keep the finger position. We suggest using a digital filter that is easy to solve these noises. This testing has demonstrated the ability of our sensor in wearable applications.

## 4. Conclusions

In this work, we have evaluated the effects of the printing-line directions (45°, 90°, 180°) on the performance of the strain sensor. The printing line at 45° improved the characteristics, especially GF, from 12.5 to 21.5, and hysteresis under 10% when compared to two other printing lines. In addition, our sensor exhibited a fast response time of 120 ms and an excellent stability of 10,000 cycles. This sensor also demonstrated its ability to detect finger bending movements in a real application. Although 3D printing is not compatible with mass production due to it being a slow process and difficult to speed up. The sensor, fabricated by FDM and CNT/TPU filament, still provides promising advantages for developing personalized garments in human motion monitoring, human-machine interface, or intelligent robots.

## Data Availability

The data used to support the findings of this study are available from the corresponding author.
